# Reversing the reversed congruency effect: directional salience overrides social significance in a spatial Stroop task

**DOI:** 10.1177/20416695241238692

**Published:** 2024-04-02

**Authors:** Yoshihiko Tanaka, Matia Okubo

**Affiliations:** 154443 Graduate School of Humanities, Senshu University, Japan; Department of Psychology, 154443 Senshu University, Japan

**Keywords:** gaze, head, spatial Stroop task, social attention

## Abstract

In a spatial Stroop task, eye-gaze targets produce a reversed congruency effect (RCE) with faster responses when gaze direction and location are incongruent than congruent. On the other hand, non-social directional targets (e.g., arrows) elicit a spatial Stroop effect (SSE). The present study examined whether other social stimuli, such as head orientation, trigger the RCE. Participants judged the target direction of the head or the gaze while ignoring its location. While the gaze target replicated the RCE, the head target produced the SSE. Moreover, the head target facilitated the overall responses relative to the gaze target. These results suggest that the head, a salient directional feature, overrides the social significance. The RCE may be specific to gaze stimuli, not to social stimuli in general. The head and gaze information differentially affect our attentional mechanisms and enable us to bring about smooth social interactions.

The direction in which others look or direct can provide a wealth of information, allowing us to infer their future actions and learn about important nearby objects. To use such information, we follow the gaze of others in the direction they are looking at (Driver et al., 1999; Emery, 2000; Oyama & Okubo, 2022). The ability to follow the gaze of others plays a crucial role in our social communication and, thus, has been studied in the field of behavioral studies as well as cognitive neuroscience (Moore & Dunham, 1995).

Marotta et al. (2018) adopted a spatial Stroop task to elucidate the unique status of gaze in social communication (also see Cañadas & Lupiáñez, 2012; Edwards et al., 2020; Fan et al., 2018; Hemmerich et al., 2022). It was originally developed to measure spatial interference in response selection (see review, Lu & Proctor, 1995). Participants responded to the target direction while ignoring its presented location in this task. Marotta et al. (2018) used eye gaze stimuli as a target in the spatial Stroop task and found that it produced a *reversed* congruency effect (RCE); reaction times (RTs) were faster when the gaze direction and its location were incongruent (incongruent trials, e.g., the right-looking target was presented in the left visual field) than when they were congruent (congruent trials, e.g., the right-looking target was presented in the right visual field). When arrows were used as targets, a spatial Stroop effect (SSE) was observed; RTs were faster for the congruent than the incongruent trials (Lu & Proctor, 1995). The reversal of the SSE of gaze is difficult to explain by general conflict control to arrow stimuli, suggesting the unique attentional mechanism of eye gaze stimuli. Subsequent research found that the RCE is modulated by facial expression (Jones, 2015; Marotta et al., 2022) and negatively correlated with the level of social anxiety (Ishikawa et al., 2021). These results accumulated the idea that the RCE suggests that eye gaze has a unique status that provides not only directional but also social information, distinguished from other directional cues such as arrows (Cañadas & Lupiáñez, 2012; Edwards et al., 2020; Hemmerich et al., 2022; Marotta et al., 2018).

There were three accounts for the RCE (Cañadas & Lupiáñez, 2012; Edwards et al., 2020; Hemmerich et al., 2022; Marotta et al., 2018). Marotta et al. (2018) proposed that the target with an inward gaze in incongruent trials made eye contact with the observer. Such eye contact facilitated spatial judgments (i.e., *eye contact hypothesis*, also see Cañadas & Lupiáñez, 2012). In contrast, Edwards et al. (2020) pointed out that the target with an inward gaze looked at the central fixation point, not the observer (Edwards et al., 2015). They proposed that joint gaze—others’ gaze attempting to establish joint attention with the observers—between the target and participants facilitated the judgment (i.e., *joint attention hypothesis*). Recently, Hemmerich et al. (2022) explained that the outward gaze targets in congruent trials distracted observers’ visual attention and delayed the responses (i.e., *joint distraction hypothesis*). Notably, all these accounts emphasize eye gaze during social interaction.

Bonventre and Marotta (2023) found the SSE when a pointing finger was used as a target in the spatial Stroop task, while they found the RCE for the gaze target (also see Dalmaso et al., 2023 for a similar result). Although gaze and pointing gestures trigger attentional orienting (Dalmaso et al., 2013), only the former stimuli may involve “special” processes related to the theory of mind (e.g., indicating the preference for objects, Ulloa et al., 2015). Bonventre and Marotta (2023) suggested that social processing specific to eye gaze reverses spatial conflict, producing the RCE, which is unique to eye gaze targets. However, to the best of our knowledge, previous studies used only a gaze or a finger as a social target in the spatial Stroop task. Thus, it is unclear how spatial interference arises when responding to other social stimuli, such as head orientation.

Head orientation is a spatial cue that seems to blend the perceptual features of the arrow and finger stimuli and the social significance of the gaze stimuli. On the one hand, heads, relative to the gaze, have more salient directional features, which are shared with the arrow and fingers. Heads are radically different from the gaze in terms of their saliency. Namely, changes in head orientation drastically alter the appearance of the face outline from the observers (Burton et al., 2009; Hermens et al., 2017; Lu & van Zoest, 2023), whereas changes in gaze direction have little effect on the facial outline because the gaze direction is defined by subtle morphological features within the eyes (e.g., the relative position of the dark iris/pupils and white sclera, Fan et al., 2018; Tanaka et al., 2023b). In Hermens et al.'s. (2017) experiments, spatial cues with various directional salience presented above and below the fixation point as either targets or distractors. Participants responded to the target direction (left/right) while ignoring the distractor. The results showed that distractors with high salient directional shapes (e.g., head, arrow, pointing gesture) interfered with judgments, but those with low salience ones (e.g., eye gaze, letters) did not. A similar outcome emerged in Burton et al. (2009). Hermens et al. (2017) suggested that directional information is processed faster for stimuli with high directional salience than those with low salience in peripheral vision. These results imply that, at least in the periphery, head orientation plays a similar role with arrows and pointing gestures in providing direction.

On the other hand, head orientation, which can work as a social stimulus, can shift the observer's visual attention to the surrounding object (Langton & Bruce, 1999; Langton et al., 2000). The attentional shift of the head is derived from the direction in which the other person is *looking*, like a gaze cue. Thus, in this respect, the head orientation may play a different role compared to an arrow or finger direction, in which they are *pointing* with their arrowhead or fingertips. Indeed, head orientation is utilized when gaze direction is obscured by shadows or sunglasses, suggesting the social role of gaze and head is conceptually close (Emery, 2000; Langton & Bruce, 1999; Langton et al., 2000). Supporting this view, gaze and head cues activate similar brain regions, including the superior temporal sulcus and fusiform gyrus (Emery, 2000). Given this evidence, the head orientation of others should play a similar role to the gaze direction when we navigate our social world. In sum, it is difficult to predict whether the head orientation produces the SSE or the RCE.

The present study used head orientation as a target in the spatial Stroop task to clarify the nature of the RCE. To achieve this aim, we used two types of targets: head and gaze (see Figure 1). The gaze target, devoid of global information about the whole face, was essentially the same as the eye gaze stimuli used by Marotta et al. (2018). Participants judged the direction in which the target was looking while ignoring its location. If the head orientation highlights the directional salience (Hermens et al., 2017), the SSE is predicted. Alternatively, if the head stimuli act similarly to gaze cues, the RCE would emerge for the head condition. On the other hand, the gaze target was essentially the same as Marotta et al.'s (2018) eye gaze stimuli and should work as control stimuli. Thus, we predicted the RCE for the gaze condition.

**Figure 1.**
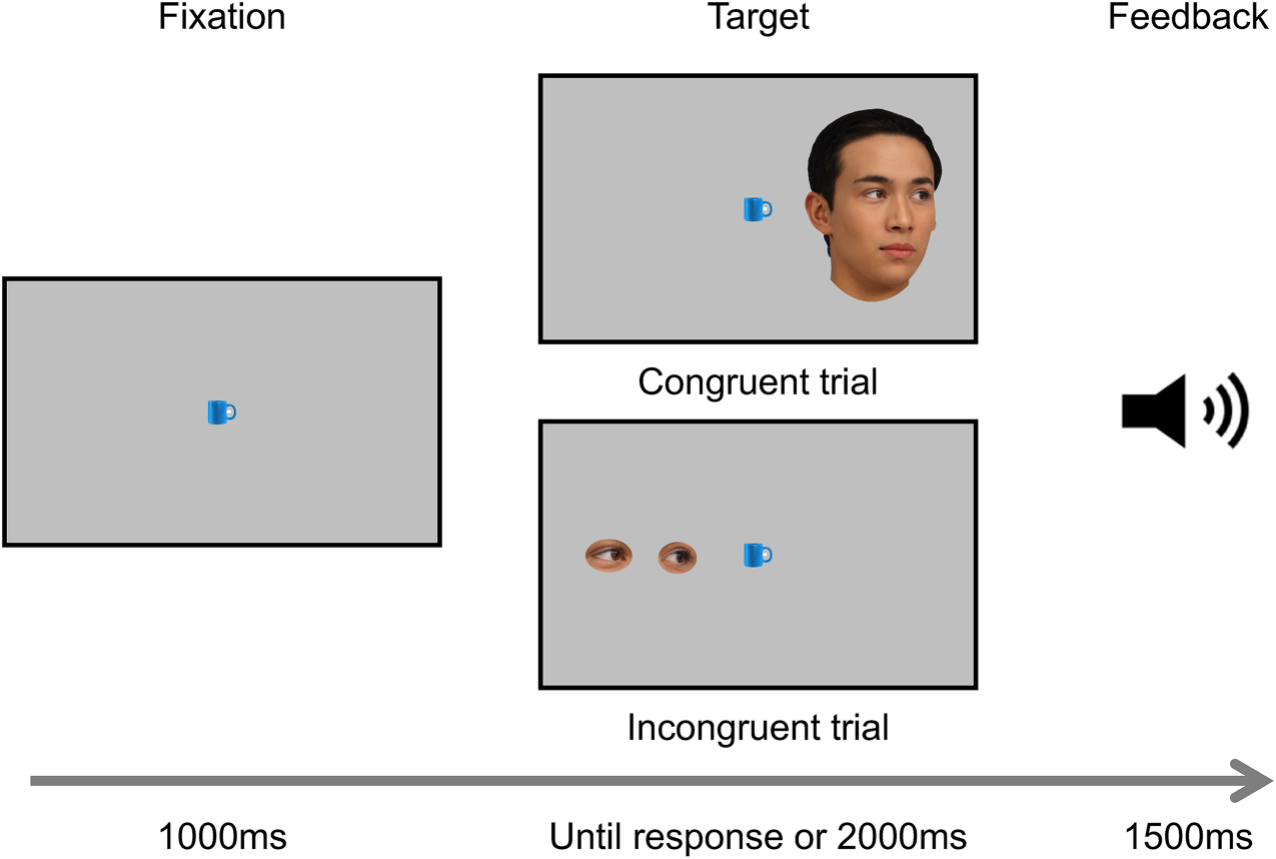
Procedures and stimuli in the spatial Stroop task.

*Note*. The examples represent a congruent trial of the target head (e.g., the right-looking target was presented in the right visual field) and an incongruent trial of the target gaze (e.g., the right-looking target was presented in the left visual field).

## Methods

### Participants

A total of 34 students (17 women and 17 men) participated in Experiment 1 (*M*_*age*_ = 21.88, *SD* = 1.55). The sample size was based on a priori power analysis conducted using G*Power 3.1 (Faul et al., 2007). Assuming an effect size of *d* = 0.50, referencing Jones (2015), a significance level of α = .05, a total sample size of 34 would provide power .80 to detect the effect. The study was approved by the Senshu University Human Research Ethics Committee (20-HP290003-2) and the participants provided written, informed consent before the experiment.

### Material

The stimuli were created referencing Marotta et al. (2018) and Edwards et al. (2020). We used two target faces, two target eye gazes, and four fixation objects, as illustrated in Figure 1. A picture of an Asian young adult male uploaded on Generated Photos (https://generated.photos/) was used as the target. There were two types of targets: head and gaze. The target heads were turned approximately 30° to the left or right (Hietanen, 1999). The gaze direction of the head target was aligned with the head orientation (see Figure 1). The head target was subtended to 7.63 deg × 10.47 deg in visual angle. The left and right eye regions were cropped from the head target to create the gaze target. The gaze target was subtended to 3.82 deg × 0.95 deg. The location and direction of the target defined the congruency condition; in congruent trials, the location and direction coincide while in incongruent trials they do not.

Four pictures of kitchen items, uploaded on CLEANPNG (https://www.cleanpng.com), were used for fixation: a frying pan, a mug, a pot, and a fork, referencing Edwards et al. (2015, 2020). The size of each fixation object was 1.43 deg × 1.43 deg on the display. PsychoPy 3.00 (Peirce, 2007) run on a MacBook Air controlled stimuli presentation, timing, and data collection. Stimuli were presented on a monitor running at a 1920 × 1080-pixel resolution. We used SONY MDR-XD150 headphones for incorrect answer feedback.

We used daily objects as a fixation point rather than a conventional cross. This was made to promote joint gaze, which is considered to elicit the RCE (Edwards et al., 2020). The presence of objects to share plays a fundamental role in joint attention (Emery, 2000). Edwards and his colleagues found that using objects as fixation points can facilitate the perception of joint gaze (Edwards et al., 2015, 2020). Therefore, we present the object as a fixation point to enhance the detectability of the RCE in the spatial Stroop task.

### Procedure

Participants were seated approximately 60 cm away from the display in a dimly lit room. The trial sequence is illustrated in Figure 1. At the beginning of each trial, one of the four fixation objects was presented in the center of the display for 1000 ms. Following the fixation object, the target was presented for 2000 ms either to the left or to the right of the object. The distance from the fixation object to the center of the gaze target was 4.77 deg. The inner-most edge of the head target was presented at the same location as the inner-most edge of the gaze. Participants judged the direction where the target was looking. They were instructed to respond as quickly and accurately as possible while ignoring the location where the target was presented. Participants pressed the “F” and “J” keys to indicate whether the target look to the left or right, respectively. If the answer was incorrect, a beeping sound (a 500 Hz tone) and the word “incorrect” in Japanese appeared for 1500 ms. The same tone along with the words “too late” in Japanese appeared for 1500 ms if a participant did not respond. The target direction and location were randomized throughout the experiment. Participants performed 16 practice trials, followed by two experimental blocks of 64 experimental trials for each condition (head condition = 64 trials, gaze condition = 64 trials). Half of the trials were congruent, whereas the other half consisted of incongruent. The order of the experimental blocks was counterbalanced among the participants.

## Results

The accuracy was very high (above 95%) and thus, was not analyzed further. Based on Marotta et al. (2018) criteria, responses faster than 200 ms (0%), slower than 1300 ms (0.07%), and incorrect responses (2.80%) were excluded from the analysis. We calculated the mean RT for four experimental conditions defined by an orthogonal combination of the target type and congruency.

**Figure 2.**
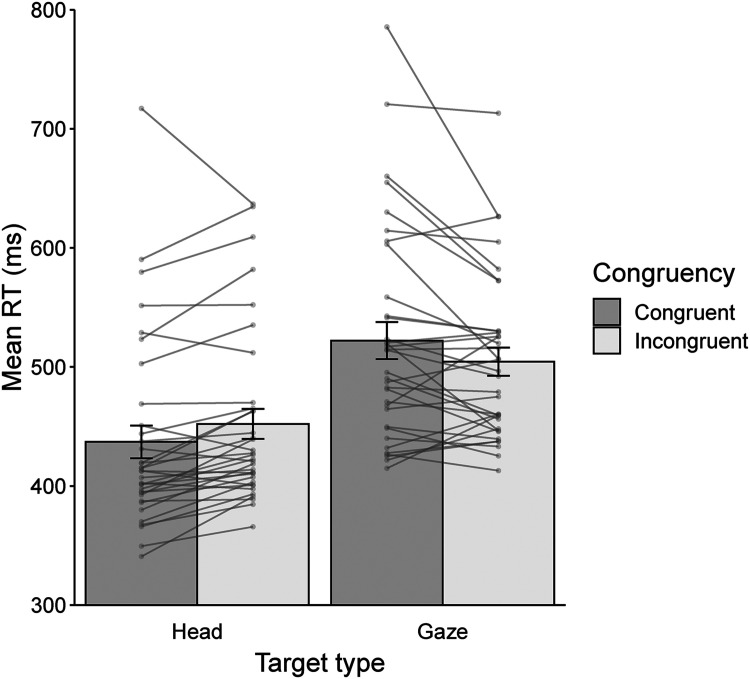
Means of RTs for the spatial Stroop task as a function of target type and congruency in Experiment 1.

*Note*. Error bars show standard errors. Plots and lines in grey represent individual data.

Figure 2 shows the means of the RT in Experiment 1. RT data were subjected to a two-factor repeated-measures ANOVA with target type (head vs. gaze) and congruency (congruent vs. incongruent). The main effect of target type was significant (*F* (1, 33) = 52.14, *p* < .001, $\eta_{p}^{2}$ = 0.61) with faster responses on the head than the gaze condition. The main effect of congruency was not significant (*F* (1, 33) = 0.03, *p* = .875, $\eta_{p}^{2}$ = 0.01). Critically, there was a significant interaction between target type and congruency (*F* (1, 33) = 22.94, *p* < .001, $\eta_{p}^{2}$ = 0.41). A simple main effect of congruency showed that RTs were significantly faster for congruent trials than the incongruent ones in the head conditions (*t* (1, 33) = 3.92, *p* < .001, *d* = 0.67). In contrast, RTs were significantly faster for incongruent trials than the congruent ones in the gaze conditions (*t* (1, 33) = 2.47, *p* = .019, *d* = 0.42).

## Discussion

The present study used head and gaze stimuli as a target in the spatial Stroop task to clarify whether the RCE occurs only when responding to eye gaze. The results demonstrated that while the RCE was replicated on gaze stimuli, the SSE appeared on the head stimuli. This pattern of the results expanded Bonventre and Marotta (2023) and Dalmaso et al. (2023), who reported the SSE on pointing gestures, accumulating evidence that the reversion of gaze may not be generalized to other social stimuli. Nevertheless, given that head orientation can function as a social cue (Emery, 2000; Langton & Bruce, 1999; Langton et al., 2000), it is surprising that the head, like gaze cues, a social stimulus, exhibits the typical SSE in the present study. However, this result is explainable if considering the directional salience of the head stimuli (Burton et al., 2009; Hermens et al., 2017; Lu & van Zoest, 2023). Head orientation has a more salient facial outline than eye gaze, which should be advantageous for identifying its direction even when in the periphery. The directionality of the head may be processed by overriding social information, leading to the SSE. The directional salience would also explain the overall facilitation of the head relative to the gaze stimuli. Participants can easily identify directional information in the head condition, resulting in overall facilitation.

One might argue that the difference in stimulus size, not the directional salience, is responsible for the present results. Indeed, the stimulus size was much larger for the head than for the gaze target. Thus, participants may have had difficulty judging the direction of the gaze target. To rule out this possibility, we conducted a follow-up experiment by adding the whole face target with a midline pose to adjust the overall stimulus size. The main results were replicated in the follow-up experiment, suggesting that the directional salience, not the stimulus size, accounted for the results (see Supplemental File).

We replicated the RCE when eye gaze was used as the target. Again, the direction of the effect was the opposite of non-social targets such as an arrow (Lu & Proctor, 1995). This replication implies that the RCE is a unique response selection toward eye gaze (Cañadas & Lupiáñez, 2012; Edwards et al., 2020; Hemmerich et al., 2022; Marotta et al., 2018). Eye gaze is a powerful social cue that contains the interests and intentions of others. The unique attentional mechanisms for eye gaze may reverse spatial interference by processing eye contact (Cañadas & Lupiáñez, 2012; Marotta et al., 2018), joint attention (Edwards et al., 2020), or joint distraction (Hemmerich et al., 2022). These results suggest that a unique attentional mechanism specialized for eye gaze, not general social stimuli, is responsible for the RCE. However, this conclusion may need some caution, given the results of the follow-up experiment (see Supplemental File); the RCE was absent for isolated-gaze stimuli, while the significant RCE emerged when embedded in a facial context with no head orientation.

As the SSE has been observed for the stimuli with high saliency (e.g., head, arrow, pointing gesture) while the RCE for those with low saliency (e.g., eye gaze), one might question if the RCE occurs due to the lower saliency of the target stimuli. In general, stimulus identification delays with the decrease of stimulus saliency. Such delayed target identification reduces spatial conflicts (Chen et al., 2022; Román-Caballero et al., 2021; Tanaka et al., 2023a). In the spatial Stroop task, location-based conflicts arise at stimulus onset and decay rapidly (Hommel, 1993). Consequently, slower identification can lead to weaker interference. For instance, Román-Caballero et al. (2021) added the complex mosaic pattern behind the arrow targets in the gaze spatial Stroop task to slow the target identification and found a reduction of the arrow's SSE. This temporal characteristic of the location-based conflicts would partially explain the results in the present study (see Tanaka et al., 2023a for theoretical details). When head stimuli were used as targets, their directional saliency accelerated the target identification. Thus, the remaining location-based conflict may interfere with directional judgments, producing the SSE. Contrary to the head stimuli, the gaze stimuli decelerated the target identification because of their lower saliency, reducing spatial conflicts. However, it is worth noting that the saliency account can explain the reduction of the SSE, not the reversal. In addition, the letter stimuli, which are included in the stimuli with low saliency (Hermens et al., 2017), can produce the SSE (Ishikawa et al., 2021). Therefore, the saliency of the targets does not fully explain the RCE, which requires further investigation.

In the present study, the gaze direction of the head stimuli was always aligned with its head orientation. As we instructed participants to judge the direction where the target is looking, without any explicit instruction for gaze or head, some participants may have responded to the gaze direction of the head stimuli. However, the overall result appeared as the SSE in head conditions. Thus, even if participants responded to the gaze direction of the head stimuli, head orientation overrode the eyes region, modulating the spatial judgments. This explanation supports the idea that the head exerts its directional salience rather than social information (Burton et al., 2009; Hermens et al., 2017; Lu & van Zoest, 2023).

Although the heads play a similar role to eye gaze in establishing joint attention (Emery, 2000; Langton & Bruce, 1999; Langton et al., 2000), head stimuli differ from gaze stimuli in some respects, such as directional saliency; head directions are obviously more salient than gaze directions (Burton et al., 2009; Hermens et al., 2017; Lu & van Zoest, 2023). Highly salient features of the head should be robust and resistant to noise, even when in the periphery. The visual system presumably utilizes such a salience of heads to obtain the available information as effectively as possible when they are in the periphery, where spatial resolution is limited. These flexible and efficient strategies of the visual system should enable us to bring about smooth social interactions.

## Supplemental Material

sj-docx-1-ipe-10.1177_20416695241238692 - Supplemental material for Reversing the reversed congruency effect: directional salience overrides social significance in a spatial Stroop task Supplemental material, sj-docx-1-ipe-10.1177_20416695241238692 for Reversing the reversed congruency effect: directional salience overrides social significance in a spatial Stroop task by Yoshihiko Tanaka and Matia Okubo in i-Perception
