# Supplementary material for: Reversing the reversed congruency effect: directional salience overrides social significance in a spatial Stroop task
Source: Iperception. 2024 Apr 2;15(2):20416695241238692. doi: 10.1177/20416695241238692 (PMC10989053; doi:10.1177/20416695241238692)
Supplement: sj-docx-1-ipe-10.1177_20416695241238692 - Supplemental material for Reversing the reversed congruency effect: directional salience overrides social significance in a spatial Stroop task [file sj-docx-1-ipe-10.1177_20416695241238692.docx]

**Supplemental material**

Experiment 1 supported the directional salience account (Hermens et al., 2017; Lu & Zoest, 2023). However, one might argue that differences in stimulus size, not the directional salience, are responsible for the results. Indeed, the stimulus size was much larger for the head than for the gaze stimuli in Experiment 1. Participants may have had difficulty judging the direction of the gaze stimuli because of its size.

Experiment 2 used three types of targets, turned head, head straight ahead, and gaze, with controlling the stimulus size. Figure 1 shows examples of the three types of targets. The turned head condition was the same as in Experiment 1 (i.e., the head turned approximately 30° to the left or right, and the gaze direction was aligned with the head orientation). The head straight ahead target, used to equate the size of the target, looked and turned straight ahead to the observer. Differently from Experiment 1, the eye region was cropped from the head straight ahead stimuli for the gaze target. The gaze direction of the head straight ahead and gaze targets were averted to either the left or right. Based on the results of Experiment 1, we predicted the SSE for the turned head conditions and faster overall reaction time. In contrast, the RCE was predicted for the head straight ahead and gaze conditions.

# Methods

The methods in this experiment were identical to those of Experiment 1.

## Participants

A total of 45 students (24 women, and 21 men) participated in Experiment 2 (*M*${}_{a}{}_{g}{}_{e}$ = 22.04, *SD* = 1.95). The sample size was based on a priori power analysis conducted using G*Power 3.1 (Faul et al., 2007). Assuming an effect size of *d* = 0.43, which was the RCE derived from Experiment 1, a significance level of $\alpha$ = .05, a total sample size of 45 would provide power .80 to detect the effect. Participants provided written, informed consent before the experiment.

## Material

We used three types of targets: turned head, head straight ahead, and gaze stimuli (see Figure 1). The turned head and head straight ahead targets are subtended 4.30 deg × 6.39 deg. The gaze target was made by cropping the eye region from the head straight ahead targets and subtended 3.82 deg × 0.95 deg. Note that the size of the eye region of all stimuli was identical across two Experiments. The stimulus set used in Experiment 1 and this Experiment was different because front-facing faces (i.e., head straight ahead targets) were not available for the face stimulus set used in Experiment 1.

The turned head stimuli were turned approximately 30° to the left or right. The gaze direction was aligned with the head orientation. The face orientation was directly ahead of the head straight ahead target while the gaze direction was averted to 30° either to the left or to the right. A head straight ahead stimulus was used to create the gaze target in this experiment.

**Figure 1.**

Example of stimulus used in Experiment 2.


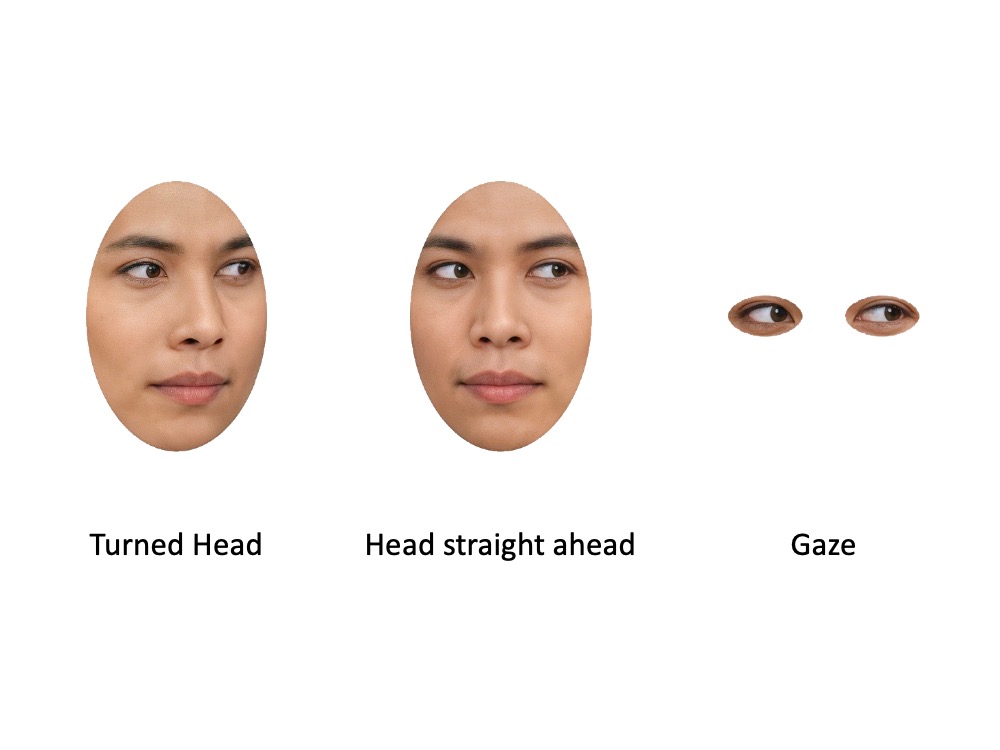


## Procedure

The procedure was identical to that of Experiment 1 except for the target location. For all target conditions, the distance from the center of the target to the fixation object was 4.77 deg. The direction and location of the target were randomly presented within the experimental block. Participants performed 16 practice trials, followed by three experimental blocks of 64 experimental trials for each condition (turned head stimuli condition = 64 trials, head straight ahead stimuli condition = 64 trials, gaze stimuli condition = 64 trials). The experiment order was counterbalanced among the participants.

# Results

The accuracy was very high (above 95%) and thus, was not analyzed further. Responses faster than 200 ms (0%), slower than 1300 ms (0.13%), and incorrect responses (2.73%) were excluded. We calculated the mean reaction time for six experimental conditions defined by an orthogonal combination of target types and congruency.

Figure 2 presents the means of the reaction times. Reaction time data were subjected to a two-factor repeated-measures ANOVA with target types (turned head vs. head straight ahead vs. gaze) and congruency (congruent vs. incongruent). The main effect of target type (*F* (2, 88) = 45.74, *p* < .001, $\eta_{p}^{2}$ = 0.51) and interaction between target type and congruency (*F* (2, 88) = 24.20, *p* < .001, $\eta_{p}^{2}$ = 0.35) were significant. The main effect of congruency was not significant (*F* (1, 44) = 1.12, *p* = .295, $\eta_{p}^{2}$ = 0.02). To clarify the main effect for target types, we conducted multiple comparisons using a Holm-Bonferroni correction test; reaction times were faster for the turned head target compared to the head straight ahead (adj. *p* < .001) and gaze targets (adj. *p* < .001). There was no significant difference between the head straight ahead and gaze targets (adj. *p* = .460).

We analyzed the simple main effect of congruency for each target-type condition to clarify their interaction. A simple main effect of congruency was significant for the turned head target with faster response for congruent trials than for the incongruent ones (*t* (44) = 5.67, *p* < .001, *d* = 0.97). In contrast, reaction times were significantly faster for incongruent trials than for the congruent ones in the head straight ahead target (*t* (44) = 3.01, *p* < .001, *d* = 0.52). No difference was found for the gaze target (*t* (44) = 0.26, *p* = .798, *d* = 0.04).

**Figure 3.**

Means of reaction times for the spatial Stroop task as a function of target type and congruency in Experiment 2.


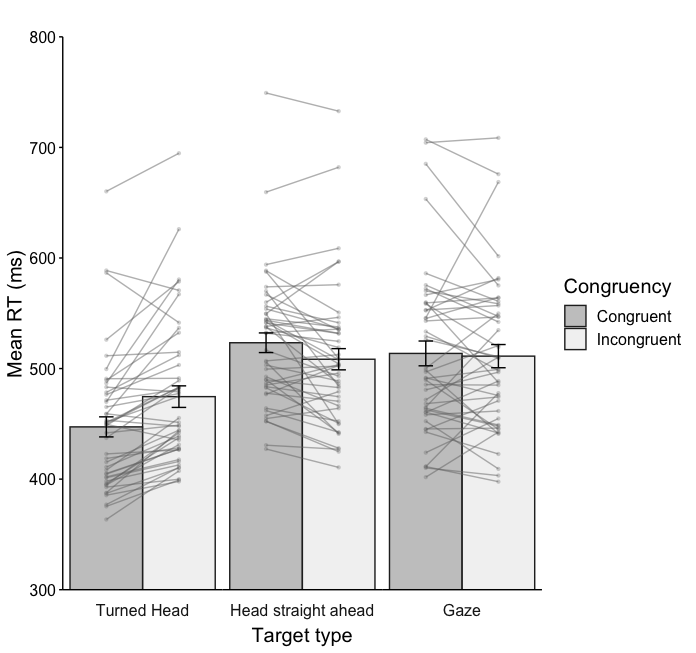


*Note.* Error bars show standard errors. Plots and lines in grey represent individual data.

# Discussion

The results of Experiment 2 replicated those of Experiment 1; the SSE was observed for the turned-head stimuli. Again, reaction times were faster for the turned head stimuli than for the head straight ahead and gaze stimuli. These results support the directional salience account; The salient facial outline of turned-head stimuli facilitates spatial judgments. Therefore, the results of Experiment 1 were not due to the stimulus difference (i.e., whole face or cropped eyes) but instead, the salient directionality of the head orientation. In contrast to the turned head stimuli condition, the head straight ahead stimuli exhibited the RCE. These results accumulate evidence that the RCE elicits only when responding to gaze stimuli and reflects the unique attentional mechanism of eye gaze (Cañadas & Lupiáñez, 2012; Edwards et al., 2020; Hemmerich et al., 2022; Marotta et al., 2018).

Contrary to Experiment 1, the RCE was unexpectedly absent in the gaze condition. Román-Caballero et al. (2021) reported that the RCE was more prominent for the whole face than for eye gaze alone. They explained that the higher segregation demand of target stimuli (i.e., gaze direction) from a complex background (i.e., facial context) eliminates the spatial interference (as shown by Hemmerich et al., 2022). Therefore, the RCE may be more stable for the whole-face target than for the cropped-eye target. We suspect that spatial interference reduced or eliminated the RCE in gaze conditions because of the lower segregation demand.

# References

Cañadas, E., & Lupiáñez, J. (2012). Spatial interference between gaze direction and gaze location: A study on the eye contact effect. *Quarterly Journal of Experimental Psychology*, *65*(8), 1586–1598. <https://doi.org/10.1080/17470218.2012.659190>

Edwards, S. G., Seibert, N., & Bayliss, A. P. (2020). Joint attention facilitates observed gaze direction discrimination. *Quarterly Journal of Experimental Psychology*, *73*(1), 80–90. <https://doi.org/10.1177/1747021819867901>

Faul, F., Erdfelder, E., Lang, A.-G., & Buchner, A. (2007). G* power 3: A flexible statistical power analysis program for the social, behavioral, and biomedical sciences. *Behavior Research Methods*, *39*(2), 175–191. <https://doi.org/10.3758/BF03193146>

Hemmerich, K., Narganes-Pineda, C., Marotta, A., Martín-Arévalo, E., Jiménez, L., & Lupiáñez, J. (2022). Gaze elicits social and nonsocial attentional orienting: An interplay of shared and unique conflict processing mechanisms. *Journal of Experimental Psychology: Human Perception and Performance, 48*(8), 824–841. <http://dx.doi.org/10.1037/xhp0001015>

Hermens, F., Bindemann, M., & Mike Burton, A. (2017). Responding to social and symbolic extrafoveal cues: cue shape trumps biological relevance. *Psychological research*, *81*, 24-42. <https://doi.org/10.1007/s00426-015-0733-2>

Lu, Z., & van Zoest, W. (2023). Combining social cues in attention: Looking at gaze, head, and pointing cues. *Attention, Perception, & Psychophysics*, *85*(4), 1021-1033. <https://doi.org/10.3758/s13414-023-02669-6>

Marotta, A., Román-Caballero, R., & Lupiáñez, J. (2018). Arrows don’t look at you: Qualitatively different attentional mechanisms triggered by gaze and arrows. *Psychonomic Bulletin & Review*, *25*(6), 2254–2259. <https://doi.org/10.3758/s13423-018-1457-2>

Román-Caballero, R., Marotta, A., & Lupiáñez, J. (2021). Spatial interference triggered by gaze and arrows. The role of target background on spatial interference. *Psicológica Journal*, *42*(2), 192–209. <https://doi.org/10.2478/psicolj-2021-0010>
